# Supplementary material for: Postconditioning by Delayed Administration of Ciclosporin A: Implication for Donation after Circulatory Death (DCD)
Source: Int J Mol Sci. 2022 Oct 25;23(21):12858. doi: 10.3390/ijms232112858 (PMC9657531; doi:10.3390/ijms232112858)
Supplement: Supplementary file 1 [file ijms-23-12858-s001.zip › ijms-1947082-supplementary.pdf]

International Journal of *Molecular Sciences*

MDPI

Postconditioning by Delayed Administration of Ciclosporin A: implication for Donation after Circulatory Death (DCD)

René Ferrera, Marie Vedère, Megane Lo Grasso, Lionel Augeul, Christophe Chouabe, Gabriel Bidaux and Delphine Baetz

SUPPLEMENTARY DATA

S1 Principle of CRC measurement (schematic diagram)

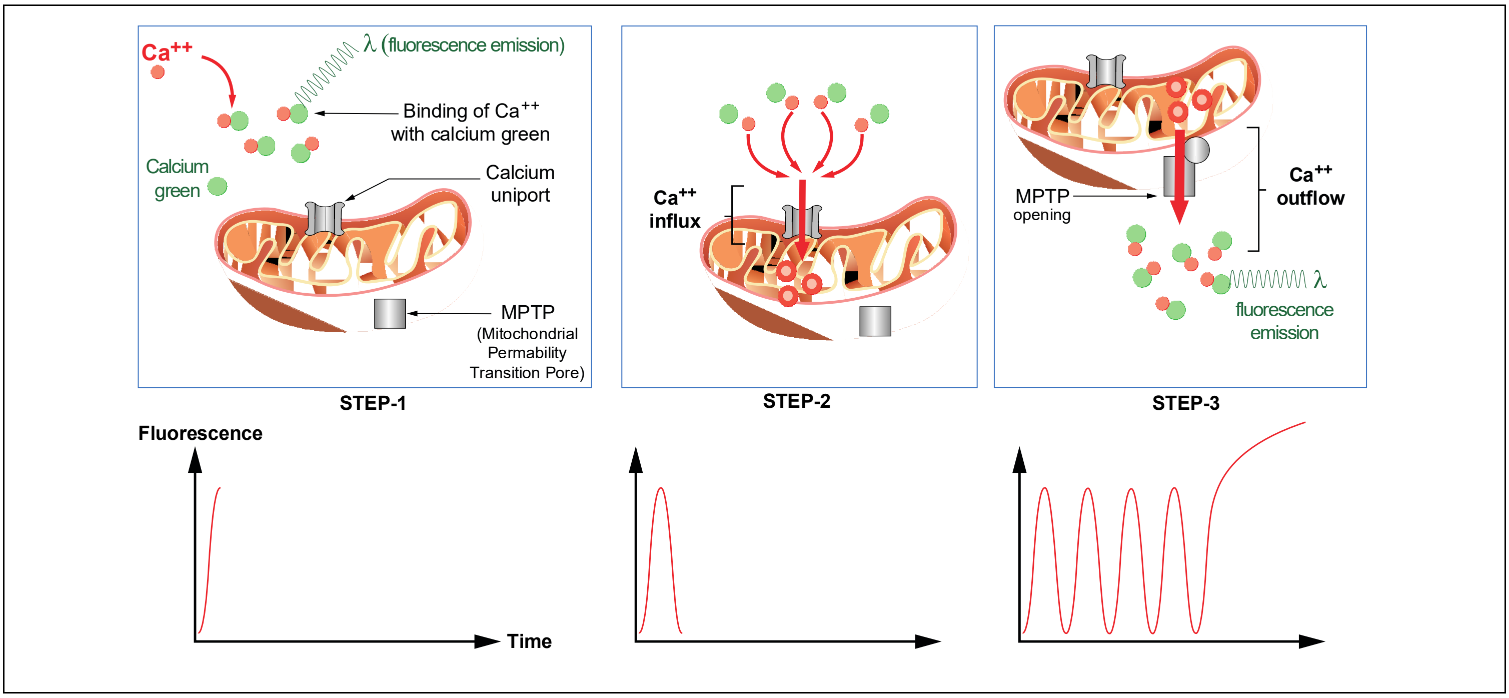

Figure S1 of supplementary data: ***method for Calcium Retention Capacity***

Calcium retention capacity (CRC) was defined here as the amount of Ca^{2+} required to trigger a massive Ca^{2+} release by isolated cardiac mitochondria.

For each of the 10 groups, mitochondria pellets were divided into four similar aliquots (250 µg proteins, determined by using the method of Bradford) Each aliquot was resuspended in 2 ml buffer (150 mM sucrose, 50 mM KCl, 2 mM KH2PO4, in 20 mM Tris/HCl, pH 7.4) and CRC was measured in the presence of:

- malate (5 mM) + glutamate (5 mM), substrates of complex I,

-. succinate (5 mM), substrate of complex II,

STEP-1. Mitochondria (250 µg proteins) were gently stirred for 2 min at 25°C. At the end of this preincubation period, 5µL $CaCl_2$ at 1mM (= pulse of 10 nmol Ca^{2+} per mg mitochondrial proteins) were added. Calcium was linked with calcium green-5N (0.5 µM, Molecular Probes™, which induced a fluorescence emission at 530 nm wavelength (and under excitation at 500 nm wavelength). Calcium pulse was recorded as a peak of fluorescence (i.e., a peak of extramitochondrial calcium concentration).

STEP-2. Ca^{2+} was then rapidly taken up by the mitochondria (Ca^{2+} influx, calcium uniport), resulting in a return of extramitochondrial calcium concentration to near baseline level. This operation was repeated every min, forming a series of fluorescence peaks.

STEP-3. Following sufficient calcium loading, extramitochondrial Ca^{2+} concentration abruptly increased (calcium outflow), indicating a massive release of the accumulated Ca^{2+} by mitochondria due to MPTP opening. The amount of $CaCl_2$ necessary to trigger this massive Ca^{2+} release (CRC) was used as an indicator of the susceptibility of mPTP to Ca^{2+} overload.

S2- Result of CRC with stimulation of complex I

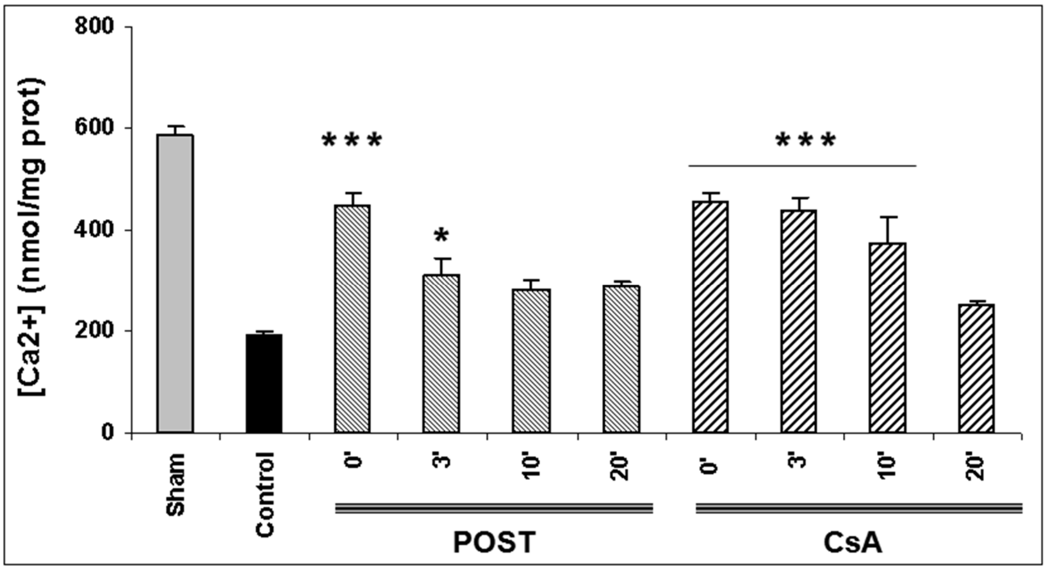

Figure S2 of supplementary data

The effect of CsA and POST on mitochondrial permeability transition pore (MPTP) opening was indirectly determined by measuring by CRC under stimulation of complex I substrates (glutamate, malate, pyruvate). In the sham group, during the presence of complex I substrates, the amount of Ca^{2+} required to open the MPTP, averaged 595±41 nmol/mg proteins. CsA treatment led to a higher CRC in groups CsA-3 and CsA-10 (p<0.001), whereas MPTP inhibition was abolished from delayed POST 10. * $p < 0.05$ and *** $p < 0.001$ vs control.

S3- ROS under stimulation of complex II

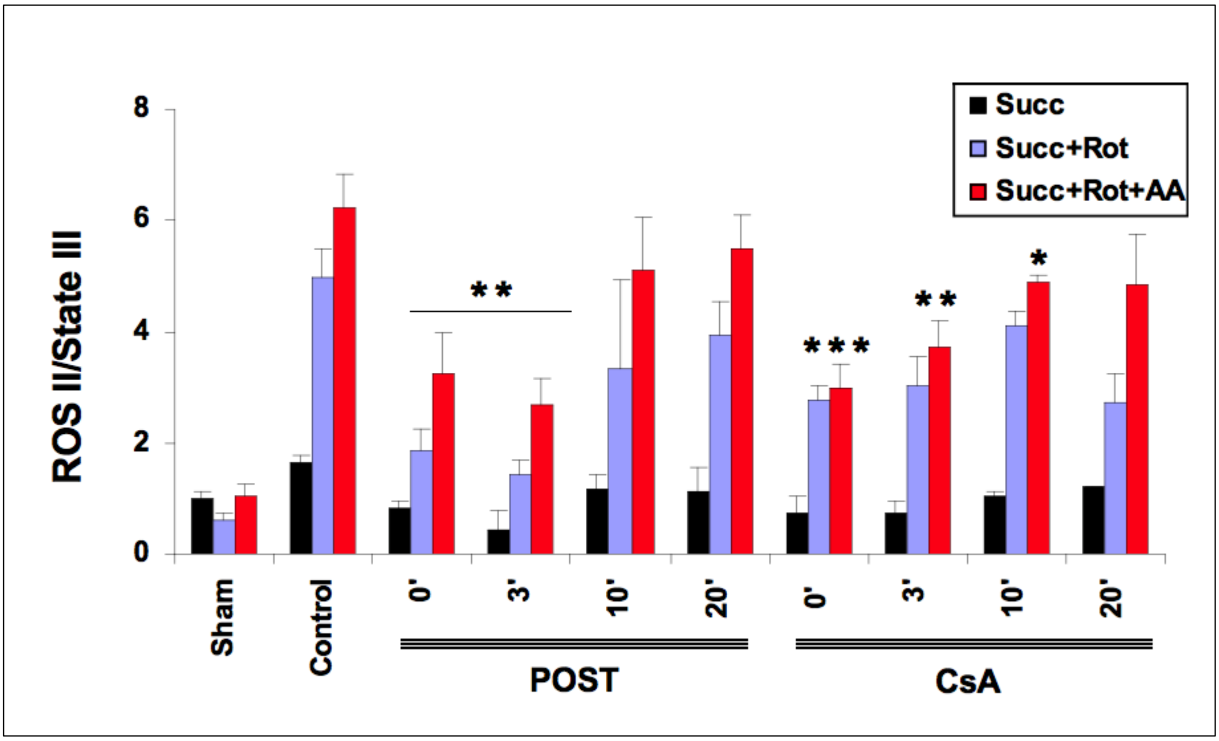

Figure S3 of supplementary data

Extramitochondrial H_2O_2 index assessed in the presence of succinate (Succ), substrate of complex II, Succ + rotenone (Rot) and Succ + rotenone + antimycin (AA). 10 minutes after the onset of reperfusion, H_2O_2 index remained lower in delayed CsA group but returned to control values in delayed POST-10 and -20.

Abbreviations: CsA for cyclosporine A and POST for ischemic postconditioning. N=6 hearts/group. *$^*p<0.05$, $^{**}p<0.01$ and $^{***}p<0.001$ vs. control.*

S4- Additive effect of CsA and Post-C

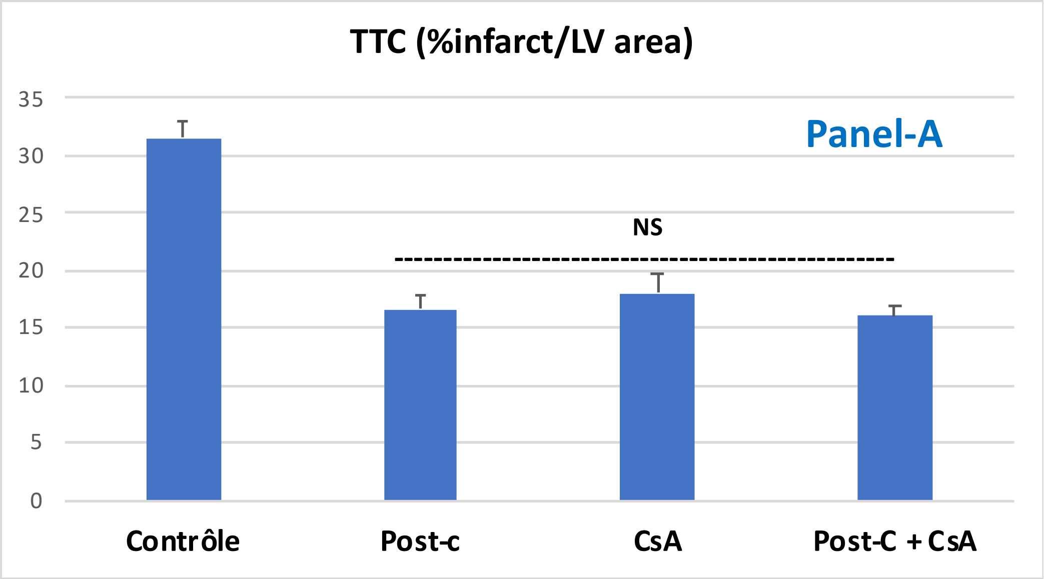

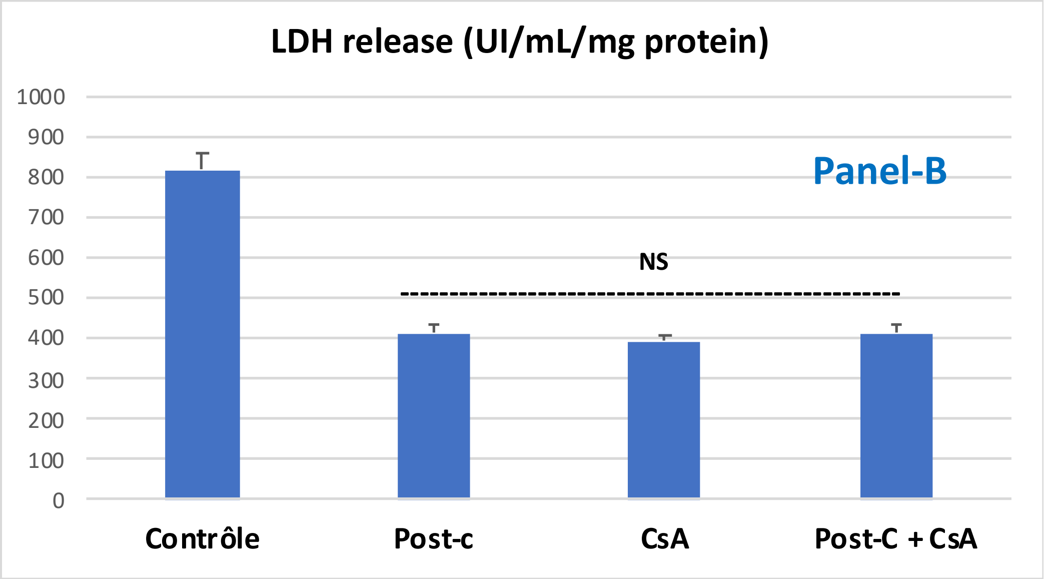

Figure S4 of supplementary data

In the parallel experiment, the potential additive effect of POST + CsA was assessed on myocardial necrosis. The same ischemia-reperfusion sequence with 40 min warm ischemia followed by 60 min reperfusion was used (see Figure-1) on isolated rat hearts. When the 2 cardioprotective maneuver were used simultaneously, no additive effect (ns = no significative) was observed regarding myocardial necrosis as assessed by TTC staining (Panel-A) or LDH leakage (Panel-B).
